# The role of ingrown hairs in persistent kerion of children: A clinical study

**DOI:** 10.1111/1346-8138.17523

**Published:** 2024-11-05

**Authors:** Qi‐Hao Yao, Hui‐Lin Zhi, Xiu‐Jiao Xia, Ze‐Hu Liu

**Affiliations:** ^1^ Department of Dermatology, Hangzhou Third People's Hospital Zhejiang University School of Medicine Hangzhou China; ^2^ Zhejiang University School of Medicine Hangzhou China

**Keywords:** dermatoscopy, ingrown hair, kerion, oral antifungal drugs, pediatrics, tinea capitis

## Abstract

Tinea capitis, a common public health problem in developing countries, has severe forms such as kerion. However, the underlying mechanisms and standard treatments for persistent cases of tinea capitis or kerion remain controversial. In this work, we investigate the ingrown hairs and corresponding treatment in persistent kerion of children. Children with persistent kerion were enrolled among 312 cases of tinea capitis at the Department of Dermatology, Hangzhou Third People's Hospital from January 2020 to June 2024. The presence of fungal infection was ascertained by direct microscopic examination under calcofluor white staining and routine culture. The structure of the ingrown hairs was observed directly by a dermatoscope, which was subsequently extracted using sterile tools. A total of six cases of persistent kerion among 312 cases of tinea capitis were enrolled. Ingrown hairs were ascertained under dermatoscopy and extracted by minor operation. Except for one patient who continued oral terbinafine, the other five cases were cured by removal alone. Ingrown hairs, induced by fungal infection, may be an aggravating factor of persistent course of tinea capitis. Our study demonstrated that the presence of ingrown hairs could be confirmed through direct dermatoscopy, and patients experienced significant improvement following removal treatment under dermatoscopy.

## 1 INTRODUCTION

Tinea capitis, an infection of the hair, follicles, and surrounding skin caused by dermatophytes, occurs with notable frequency in children, including those who are not immunocompromised, with a particular prevalence among preschool‐aged children.^1^ Although the spectrum of pathogens varies significantly with geography and over time, tinea capitis continues to be a global public health issue, particularly in developing countries where sanitation and nutrition may be inadequate.^2^ Kerion, a severe inflammatory form of tinea capitis, can result in scarring alopecia and extensive tissue damage, in addition to common symptoms such as erythema, scaling, and abscess formation.^3^

Persistent kerion exhibits more extensive involvement and increased severity in the infrequently reported cases of tinea capitis that are refractory to treatment, and managing cases resistant to systemic antifungal agents poses a significant challenge. In this process, ingrown hairs may play an important role. Ingrown hairs, also named pseudofolliculitis barbae (PFB), can act as foreign bodies to cause local inflammations.^4^, ^5^ The erythema papulopustular lesions mainly involves the beard, neck, submental, and groin areas.^6^ In fact, ingrown hairs can occur in any area where traumatic hair removal has been performed, but reports of scalp involvement are rare, especially in infections.^7^ Clinical practice in our patients suggested that ingrown hairs might be a contributing factor to the prolonged duration and suboptimal outcomes in persistent cases of tinea capitis, particularly in kerion. Further research into the cause and progression of this condition could enhance prevention and treatment strategies for tinea capitis that is unresponsive to conventional therapies.

This prospective study evaluated persistent kerion cases among children with tinea capitis at our hospital, documenting six instances that showed limited improvement with standard antifungal therapy and were subsequently treated effectively with ingrown hair removal. The study also discussed the potential role of ingrown hairs in superficial fungal infections affecting the hair.

## 2 MATERIALS AND METHODS

From January 2020 to June 2024, 312 children diagnosed with tinea capitis were enrolled. The criteria for the diagnosis of tinea capitis included: (1) age younger than 18 years; (2) presence of fungal elements confirmed by microscopic examination of scalp samplings or hair; and (3) routine fungal culture showing positive results; if the culture was negative, then two positive results of microscopic examination conducted no more than 15 days apart associated with typical clinical symptoms.

Among these cases, persistent kerion was further defined by two more criteria. Kerion was defined as typical inflammatory swelling plaques caused by fusion of follicular pustules, with manifestations of pus, crusts, and alopecia on the head. Persistent ones were defined as those that showed no significant improvement or even progression after at least 4 weeks of treatment with the standard dose of systemic antifungal therapy. Their detailed information is provided in the results.

All specimens of suspected tinea capitis were operated for fungal identification according to our standardized protocol. In brief, broken hair, dander, and abscesses were collected for direct and fluorescent staining microscopic examination. At the same time, specimens were inoculated onto Sabouraud glucose agar with chloramphenicol and cycloheximide and incubated at 25°C for 2 to 4 weeks. The identification of species was mainly based on morphological identification, such as the macroscopic characteristics of Sabouraud dextrose agar culture and the microstructure of potato glucose agar slide culture, combined with clinical appearance and physiology.

In children with persistent kerion, suspected ingrown hairs were directly observed using a portable dermatoscope. Scars and affected skin were removed with a sterile scalpel and needle to confirm the presence of ingrown hairs and removal if necessary. Two videos depicting the specific procedural steps are included as supporting information for reference (Video S1 and S2). After removal, similar identification methods, including fluorescent staining and microscopic examination, along with routine culture as previously described, were employed to ascertain whether the fungal components within the ingrown hairs matched those found in broken hair or dandruff from the scalp. Confirmed cases with ingrown hairs were evaluated at regular follow‐up. The removal treatment was continued until the lesions were completely resolved, and all potential ingrown hairs had been removed, concurrent with achieving three consecutive negative mycological results.

This study used data recorded anonymously in a database (the mycology laboratory information management system and medical information system of our hospital) to select the cases and follow their course. Written informed consents were obtained from the parents of the child patients for the use of figures. This study was approved by the medical ethics committee at the Hangzhou Third People's Hospital (number 2020KA005).

## 3 RESULTS

Among the 312 cases of tinea capitis in children, six cases were finally defined as persist kerion according to our standard. General information of each case is presented in Table 1. The detailed cases are below.

**TABLE 1:** General information of the 6 cases.

Case	Age (year)	Sex	Species of pathogen	Initial antifungal drug	Duration of antifungal treatment before being identified as persistent kerion	Number of removal treatment	Year of infection
1	6	Female	*Trichophyton mentagrophyte*	Terbinafine	4 months	2	2020
2	7	Female	*Microsporum canis*	Itraconazole	5 weeks	3	2021
3	5	Male	*Trichophyton mentagrophyte*	Terbinafine	1.5 months	3	2022
4	5	Male	Unclear	Itraconazole	2 months	2	2022
5	4	Female	Unclear	Terbinafine	2.5 months	2	2023
6	7	Male	*Trichophyton rubrum*	Terbinafine	2 months	3	2023

### 3.1 Patient 1

**FIGURE 1:**
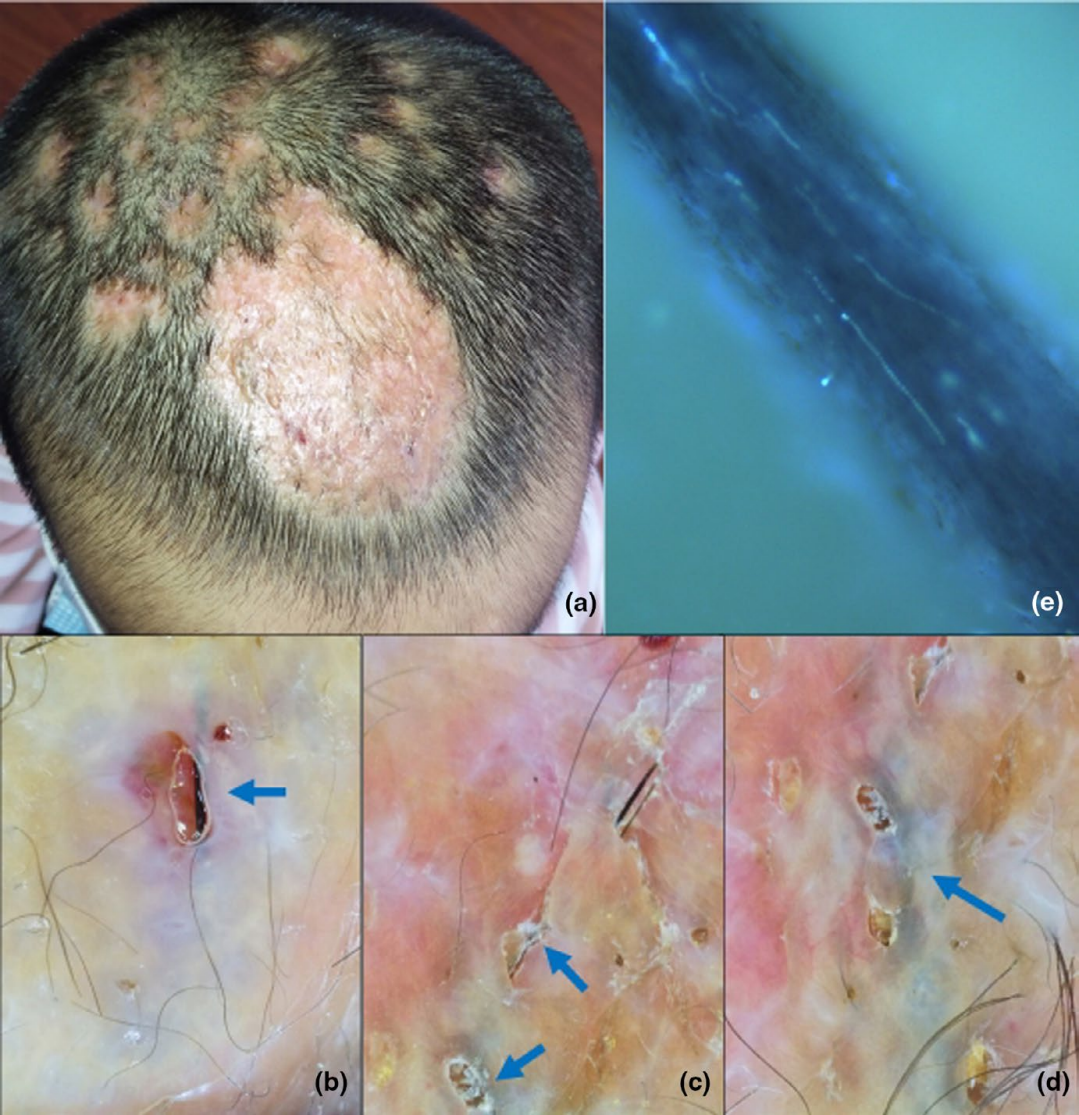
Clinical and dermatoscopic presentations of patient 1. (a) Scarring alopecia and inflammatory swelling plaques on her scalp. (b–d) Black linear structures beneath the scars, confirmed to be ingrown hairs. (e) Fluorescence microscopy detected endothrix hair invasion (×400).

A 6‐year‐old girl presented with a 2‐month history of alopecia and swollen plaques on her scalp. She received no treatment before being diagnosed with kerion caused by *Trichophyton mentagrophyte*. Oral terbinafine 125 mg/day for 4 months and prednisone treatment for 14 days showed poor response. Physical examination revealed scarring alopecia and inflammatory swelling plaques with black linear structures on the scalp (Figure 1a). Dermatoscopic evaluation identified these structures as ingrown hairs, which were successfully removed under dermatoscopic guidance using sterile instruments, including a needle and tweezers (Figure 1b–d). Endothrix hair invasion was confirmed by fluorescent microscopy with calcofluor white staining (Figure 1e). Following the removal, oral terbinafine was continued for additional 2 weeks, which led to a response in the lesions and negative mycological results. This case was the only patient who received continued systemic antifungal medication during the removal of the ingrown hairs. Subsequent follow‐up revealed no signs of recurrence, and the patient made a full recovery.

### 3.2 Patient 2

**FIGURE 2:**
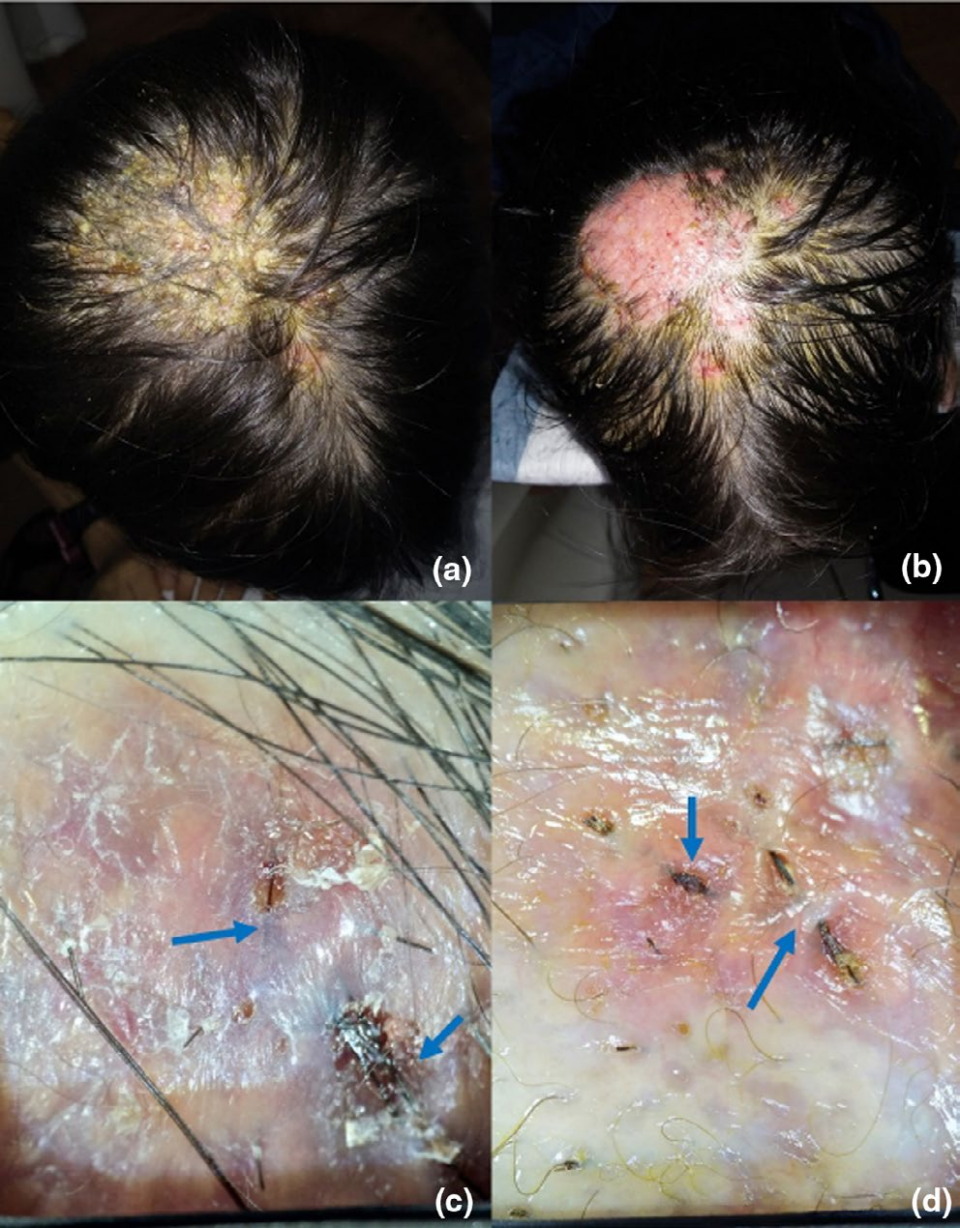
Clinical and dermatoscopic presentations of patient 2. (a) Yellow thick scales attached to both hair and scalps, with scattered erythema papula. (b) After scale removal, swollen red plaques could be seen with pustules and hemorrhagic spots. (c, d) Ingrown hairs could be seen beneath the skin.

A 7‐year‐old girl presented with yellow, thick scales on both the hair and scalp, accompanied by scattered erythematous papules for 2 months (Figure 2a). Swollen, red plaques with pustules and hemorrhagic spots were visible beneath the scales (Figure 2b). Alopecia also existed. The fungal culture revealed *Microsporum canis*. However, 5 weeks of oral itraconazole combined with prednisone showed no improvement in the swelling and pustules. Dermatoscopic evaluation showed similar black lines with the same fungal involvement (Figure 2c,d). Over the course of one and a half months, three consecutive ingrown hair removals were performed without additional antifungal medications. Two months after the last removal, the lesions basically returned to normal, with new hairs visible (Figure 7a). Under dermatoscopy, aside from a small amount of yellow scale, the appearance of the hair was basically normal (Figure 7c).

### 3.3 Patient 3

**FIGURE 3:**
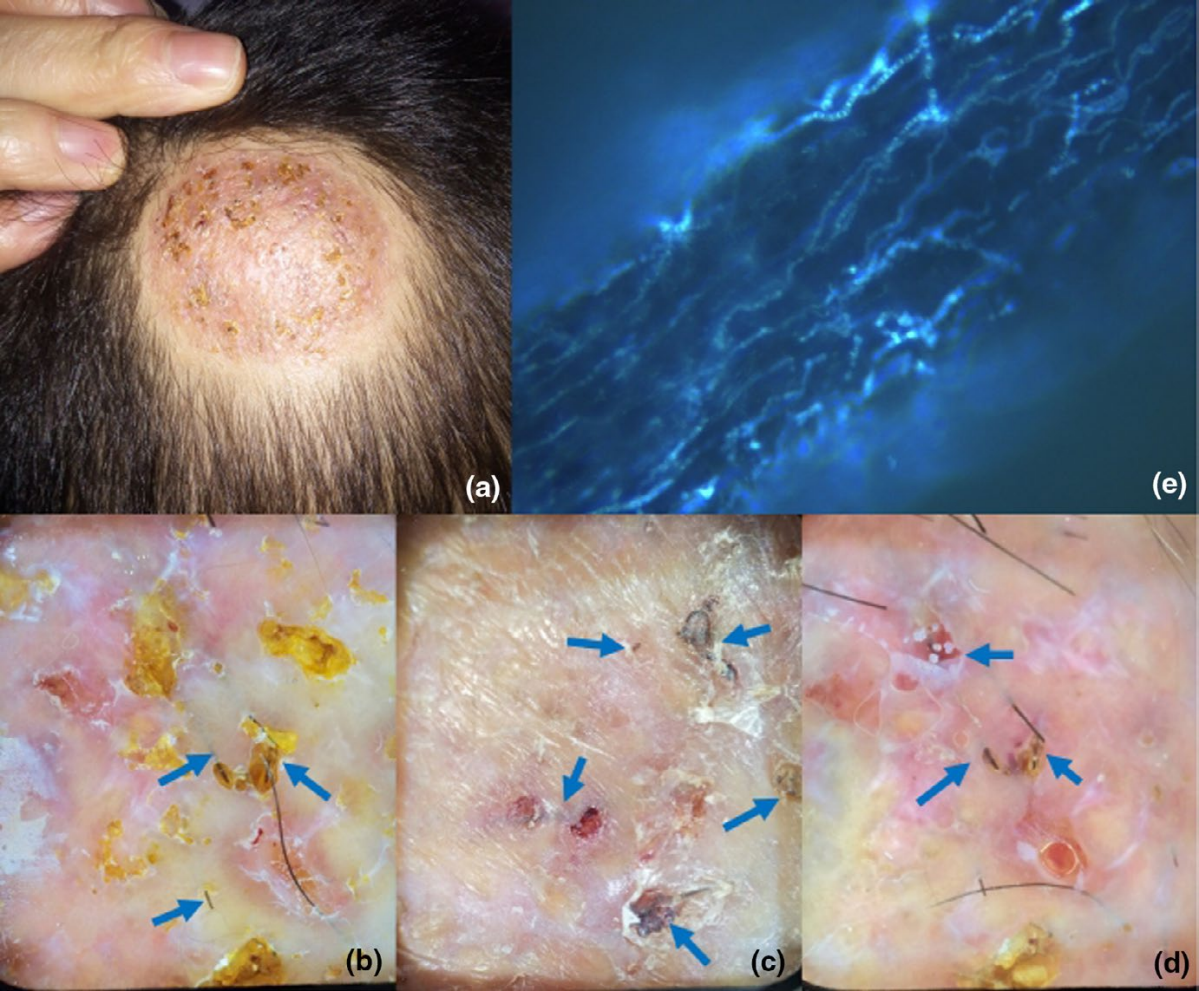
Clinical and dermatoscopic presentations of patient 3. (a) Swelling responded poor to treatment, with newly emerging yellow scales. (b–d) Ingrown hairs could be seen on the background of scales and hemangiectasis. (e) A large number of mycelia could be seen under fluorescent stain in ingrown hairs taken from the scalp (×400).

A 5‐year‐old boy presented with erythema and an isolated pustule on his right scalp, which had persisted for 1 week. The lesions showed no improvement with oral cephalosporin treatment, developing into inflammatory plaques with pustules and purulent discharge over the following week. The fungal culture identified *T. mentagrophyte*. A diagnosis of kerion was made with confirmed endothrix hair invasion. However, the swelling and endothrix hair invasion showed no improvement with oral terbinafine 125 mg/day over the next month and a half, and new yellow scales emerged (Figure 3a). Dermatoscopic examination revealed scattered subcutaneous black linear structures (Figure 3b–d). Subsequent microscopic examination confirmed a large amount of endothrix mycelium in ingrown hairs under calcofluor white staining (Figure 3e). Terbinafine was discontinued. After 1 month of follow‐up, the patient's lesions stopped progressing with removal treatment two times. Recovery was observed 2 months later, with hair loss and new hair coexisting.

### 3.4 Patient 4

**FIGURE 4:**
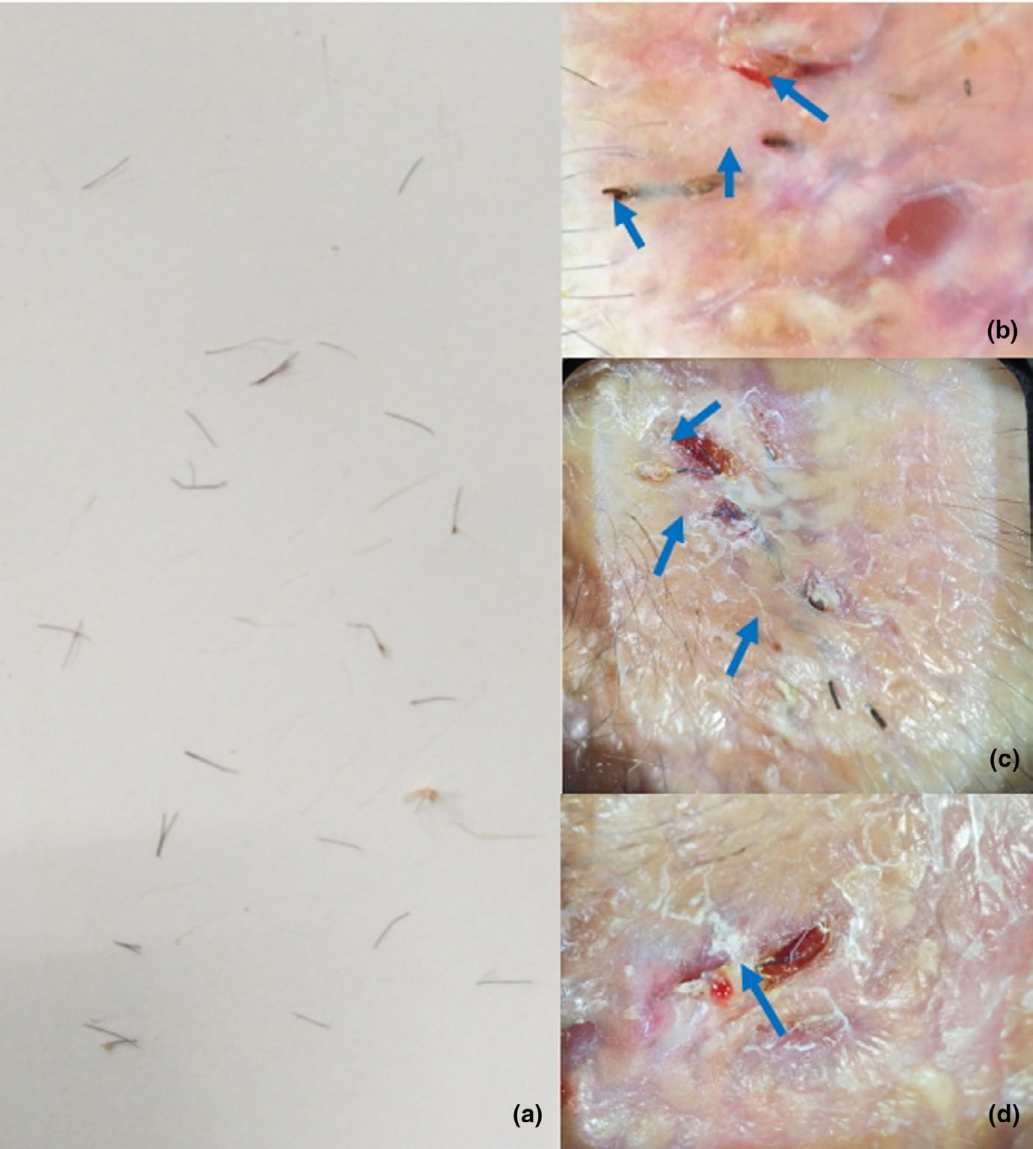
Clinical and dermatoscopic presentations of patient 4. (a) Ingrown hairs taken from the scalp, placed on white paper. (b–d) Ingrown hairs could be seen on the background of superficial scarring and scattered hemorrhage.

A 5‐year‐old boy presented with scales and pustules on his scalp for more than 1 month. His parents reported contact with dogs, and the local hospital had performed surgical resection with topical antibiotic treatment, showing poor effect. Scattered ulcerations and swollen plaques were observed, but routine culture failed to confirm the diagnosis. Kerion was diagnosed based on positive fluorescent microscopic examination and clinical manifestations. Oral itraconazole 100 mg/day for 2 months resulted in slight improvement. However, deformed ingrown hairs (Figure 4a) and scattered bleeding were still evident upon dermatoscopic examination. The hair on the scalp surface and the removed ingrown hairs (Figure 4b–d) demonstrated similar characteristics upon direct microscopic examination with calcofluor white staining. The visible ingrown hairs were removed, and itraconazole was discontinued. Half a month later, a small number of ingrown hair structures were removed once more within significantly reduced lesions. After a subsequent month, there was a significant improvement in the affected lesion area.

### 3.5 Patient 5

**FIGURE 5:**
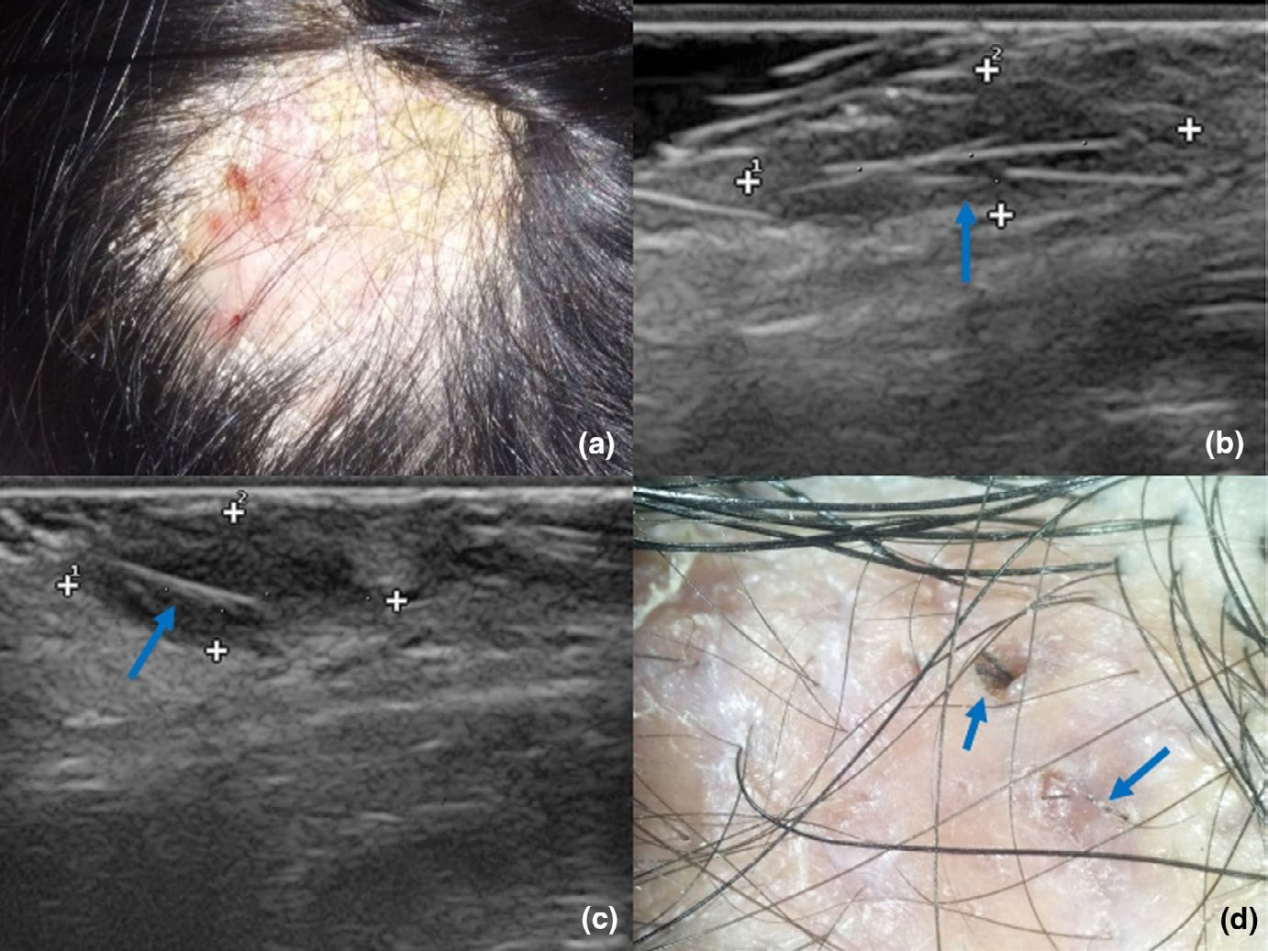
Clinical and dermatoscopic presentations of patient 5. (a) Yellow scales, scattered hemorrhagic spots, and hair loss remained unchanged after treatment. (b, c) Hyperechoic structures resembling hair could be seen in hypoechoic structures under the skin. The substance was subsequently identified as irregularly growing ingrown hairs. (d) Ingrown hairs were visible, piercing the surface of skin.

A 4‐year‐old girl presented with erythema, swelling, and exudation on her scalp for 3 days. The lesions developed into swollen plaques within 1 week, surrounded by scattered pustules. A diagnosis of kerion was made based on her clinical manifestations and positive results from fluorescence microscopic examination. Routine culture failed to confirm the pathogen. After two and a half months of antifungal treatment with terbinafine 125 mg/day, the swelling improved, but numerous yellow scales, scattered hemorrhagic spots, and hair loss persisted (Figure 5a). B‐ultrasonography revealed hyperechoic hair–like shadows within a superficial hypoechoic mass (Figure 5b,c), and similar black linear structures were observed under dermatoscopy (Figure 5d). The removal treatment was performed twice within half a month, and oral terbinafine was discontinued. During the follow‐up visit 1 month later, no new ingrown hairs were detected upon dermatoscopic examination, and there was further improvement in the lesions.

### 3.6 Patient 6

**FIGURE 6:**
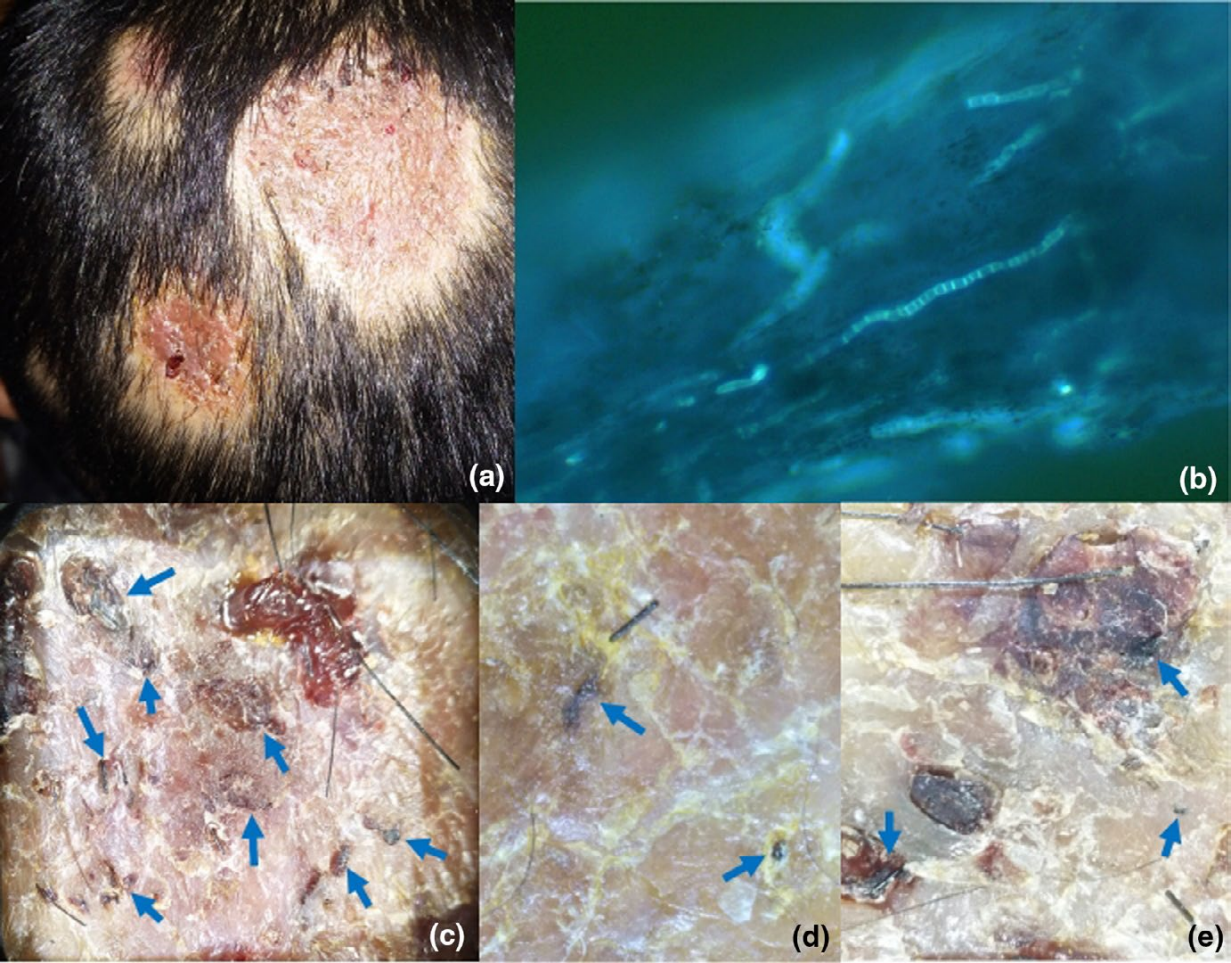
Clinical and dermatoscopic presentations of patient 6. (a) Atrophic plaques and multiple scattered subcutaneous abscesses. (b) Mycelia on the surface of the ingrown hairs under fluorescent stain (×400). (c–e) Multiple ingrown hairs beneath the scarring, with surrounding hemorrhage and blood scab.

**FIGURE 7:**
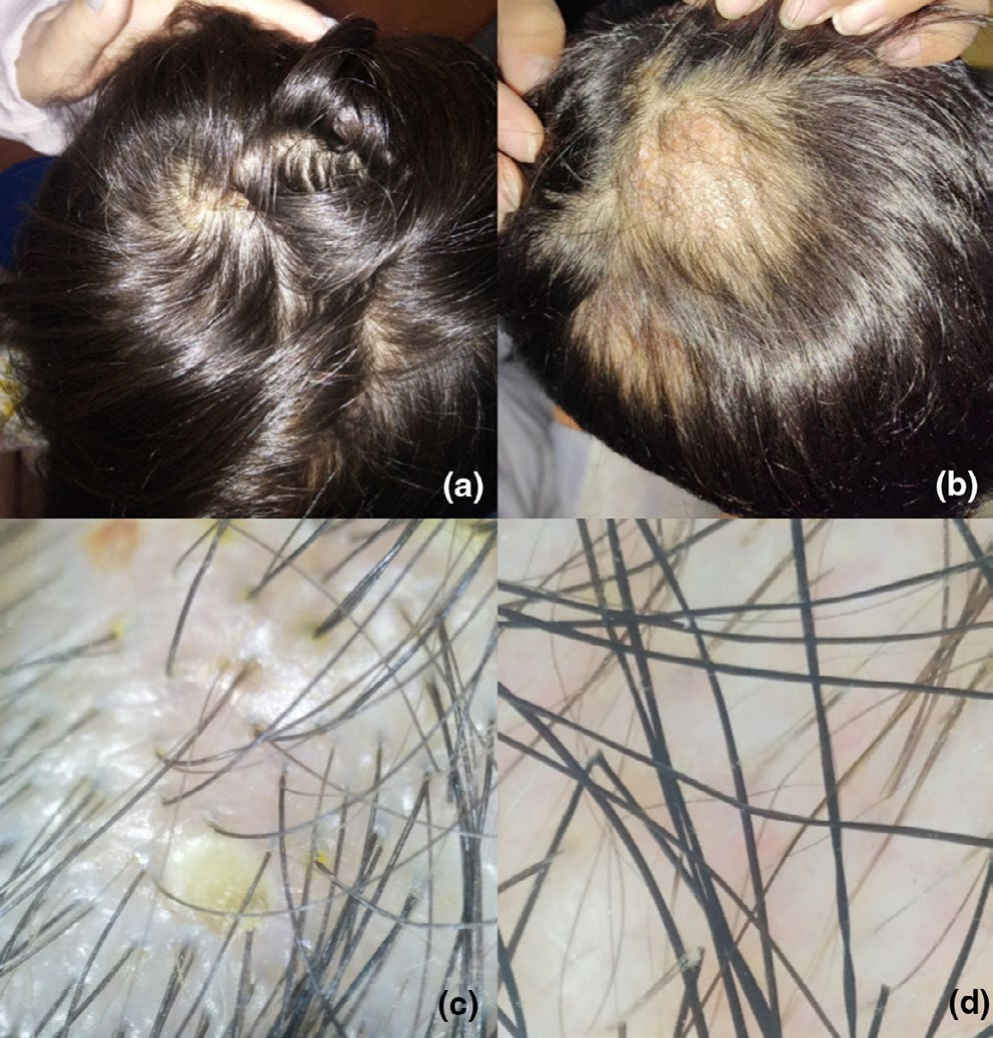
Clinical and dermatoscopic presentations of cured patients 2 and 6. (a) Yellow scales and swollen red plaques disappeared, with new hairs growing (Patient 2, about 2 months after the last removal of ingrown hairs). (b) The atrophic plaques significantly improved with no obvious pustules. New hairs could be seen on the original lesions. (Patient 6, about 1 month and a half after the last removal of ingrown hairs.) (c) Aside from a small amount of yellow scale, the hair morphology appeared essentially normal under dermoscopy (patient 2). (d) Part of the scalp was slightly red under dermatoscopy, with the new hair basically normal (patient 6).

A 7‐year‐old boy presented with erythema, subcutaneous abscesses, and scales on his scalp for 10 days. During the past 3 days, the lesions exhibited increased swelling and palpable abscess formation. A diagnosis of kerion was confirmed based on the detection of *Trichophyton rubrum* and clinical manifestations. Treatment with oral methylprednisolone 8 mg/12 h and terbinafine 125 mg/day for 2 weeks significantly reduced local swelling. However, continuing terbinafine alone for additional 6 weeks yielded no further improvements. Physical examination revealed atrophic plaques, significant alopecia, and multiple scattered subcutaneous abscesses (Figure 6a). Black linear structures were also observed under dermatoscopy (Figure 6c–e), which had fungal components consistent with *T. rubrum* after removal (Figure 6b). Visible ingrown hairs were removed at 2‐week intervals with discontinued terbinafine. The removal treatment was performed three times. A month and a half after the last treatment, no new ingrown hairs were observed. The atrophic plaques improved significantly (Figure 7b), and part of the scalp was slightly red under dermoscopy, with the new hair basically normal (Figure 7d).

## 4 DISCUSSION

Tinea capitis, a dermatophytes infection affecting scalp and hair, is particularly prevalent among children, especially those who are preadolescent.^1^ In China, the average age of onset is about 4 years old, which is in line with previous literature indicating an onset range of 1 to 5 years old.^8^, ^9^ The high incidence in children may be attributed to several factors: an immature skin barrier function, insufficient secretion of fatty acids that inhibit fungal growth, a strong curiosity, and a lack of hygiene awareness, all of which can lead to easier exposure to infection sources.^10^

Clinically, tinea capitis could be categorized into different subtypes based on the causative fungal species and the patient's immune response. The genotypes of dermatophytes influence the activity levels of enzymes such as keratinase, collagenase, and elastase, potentially leading to varying degrees of infection severity. However, the precise correlation between the activity of these secretases and the severity of the infection has yet to be established.^11^

It is widely accepted that animal‐derived pathogens, with *M. canis* being the most common, are more prone to induce severe inflammatory reactions such as kerion, characterized by inflammatory follicular papules and raised masses encrusted with pustules and thick scabs.^12^ Untreated kerion can result in permanent scarring alopecia.^13^ Although previous reports have documented cases of kerion that show poor responses to conventional systemic antifungal treatments, regardless of whether in children with normal or congenital immunodeficiency, the potential role of ingrown hairs as a causative factor has not been previously discussed.^14^, ^15^ We searched the relevant databases using the terms “ingrown hairs” and “tinea capitis” or “kerion,” finding no previous literature describing the condition. Although the infection species, affected range, and duration of disease varied among the six patients with persistent kerion in our study, poor response to standard oral antifungal drugs and the presence of ingrown hairs were consistent.

Ingrown hairs, historically referred to as PFB or pseudofolliculitis of the beard, usually presents as straight, creeping, or U‐shaped ingrowing hairs beneath painful or itchy erythema papuloid skin.^4^ The development of ingrown hairs is associated with inflammation induced by the foreign body reaction in traumatic hair removal or the inflammation of hair follicles in cicatricial alopecia.^5^, ^16^, ^17^ The pathogenesis is multifactorial, encompassing the structural and property characteristics of hair follicles and hair, genetic susceptibility attributable to polymorphisms of Ala12Thr in KRT75 gene, and personal hygiene.^17^ As a result, the hair does not grow perpendicularly to the skin's surface as normal; instead, it grows beneath the skin or pierces through the epidermis, forming a “pseudofollicle structure” or “transfollicular penetration”.^18^, ^19^ However, the role that acute inflammation or infections play in the development of ingrown hairs has not received corresponding attention.^20^

The hair and nails with superficial fungal infections closely resemble the morphological abnormalities, including broken and rough hair shafts, as well as loosely attached and hypertrophic nails, in normally developing mice with mutations of Krt75, a member of the keratin family.^21^ Just as the role Krt75 plays in the pathogenesis of PFB, fungal infections may promote the formation of ingrown hairs through a similar mechanism, namely as rough and sharp ends close to the scalp and curly configurations of hairs.^22^, ^23^, ^24^, ^25^ These manifestations can be primarily evaluated using dermatoscopy: the former includes black spot signs, broken hairs, and cigarette‐shaped hair, while the latter includes comma, corkscrew, zigzag, and bent hairs.^26^ The nonspecific keratin structure destruction caused by keratinophilic fungi, explaining the hair morphological changes, has also been confirmed by scanning and transmission electron microscopy: disrupted and loosely packed ends in cigarette‐shaped hairs; bent hair shafts; asymmetrically disrupted cuticle layers; and the absence of the normal ratcheted appearance in corkscrew hairs.^27^, ^28^, ^29^ Taking into account the existence of these phenomena, ingrown hairs caused by keratin structural abnormalities may also exist in other types of tinea capitis rather than our definition of “persistent kerion,” with different degrees.

Integrating the discussion with our clinical experience, we conclude that ingrown hairs, arising from fungal infections, play a role in the dissemination of infection and the development of resistance to treatment. Several parts are involved: the abnormal structural traits predispose to ingrown hairs formation, facilitating the local spread of pathogens through subcutaneous and transcutaneous penetration. This process effectively “reimplants” the affected hairs in an erroneous growth trajectory, contrary to the natural.^27^ At the same time, it can be viewed as “primary infectious foreign bodies,” which intensifies local inflammation, leading to the formation of secondary lesions and hindering the penetration of antifungal agents to the site of infection.^30^ Within this process, ingrown hairs act as a potential fungal reservoir due to their unique location and the inflammatory milieu, thereby impeding the efficacy of conventional systemic antifungal treatments.

A minor surgical procedure involving the use of a dermatoscope and sterile instruments to locate and remove ingrown hairs containing fungal elements offers a straightforward and cost‐effective treatment option, particularly for patients whose conditions persist or worsen under conventional therapy, as in our cases. This method can decrease the fungal burden, facilitate the identification of secondary lesions, and circumvent the potential side effects associated with prolonged systemic antifungal treatment, including liver dysfunction and systemic adverse reactions.^31^ The definition of “ingrown hairs” should encompass a broader spectrum of conditions, extending beyond PFB or creeping hair to specific cases associated with specific causes, including fungal infections or other acute inflammations.

To our knowledge, this is the first report describing the link between ingrown hairs and fungal infections, suggesting that early detection and removal of ingrown hairs has considerable curative effects on persistent kerion. Nonetheless, large sample research is required to assess the similarities and differences associated with ingrown hairs among different cases. Specifically, examining the overall efficacy of the “removal treatment” and developing standardized protocols might enhance our current understanding and approach to kerion treatment.

## FUNDING INFORMATION

This work was supported by the Hangzhou Science and Technology Bureau, China (grant number 202004A17) and Hangzhou Health Science and Technology Plan, China (grant number Z2024015).

## CONFLICT OF INTEREST STATEMENT

The authors have no relevant financial or non‐financial interests to disclose.

## ETHICS STATEMENT

This study was approved by the medical ethics committee at the Hangzhou Third People's Hospital (number 2020KA005).

## CONSENT

The removal of the ingrown hairs and publication of the images was obtained with consent of the patients' parents.

## Supporting information


Video S1 and S2.
