# Supplementary material for: The role of ingrown hairs in persistent kerion of children: A clinical study
Source: J Dermatol. 2024 Nov 5;52(1):35–42. doi: 10.1111/1346-8138.17523 (PMC11700942; doi:10.1111/1346-8138.17523)
Supplement: Supplementary file 1 — Video S1 and S2. [file JDE-52-35-s001.zip › jde17523-sup-0001-Video captions.docx]

**Video captions**

**Supplement 1 and 2**

The removal of ingrown hairs under dermatoscopy using sterile tools, with no obvious bleeding.
